# Maternal Polybrominated Diphenyl Ether (PBDE) Exposure and Thyroid Hormones in Maternal and Cord Sera: The HOME Study, Cincinnati, USA

**DOI:** 10.1289/ehp.1408996

**Published:** 2015-04-17

**Authors:** Ann M. Vuong, Glenys M. Webster, Megan E. Romano, Joseph M. Braun, R. Thomas Zoeller, Andrew N. Hoofnagle, Andreas Sjödin, Kimberly Yolton, Bruce P. Lanphear, Aimin Chen

**Affiliations:** 1 Division of Epidemiology, Department of Environmental Health, University of Cincinnati College of Medicine, Cincinnati, Ohio, USA; 2 Child and Family Research Institute, BC Children’s and Women’s Hospital and Faculty of Health Sciences, Simon Fraser University, Vancouver, British Columbia, Canada; 3 Department of Epidemiology, Brown University School of Public Health, Providence, Rhode Island, USA; 4 Department of Biology, University of Massachusetts Amherst, Amherst, Massachusetts, USA; 5 Department of Laboratory Medicine, University of Washington, Seattle, Washington, USA; 6 Division of Laboratory Sciences, National Center for Environmental Health, Centers for Disease Control and Prevention, Atlanta, Georgia, USA; 7 Division of General and Community Pediatrics, Department of Pediatrics, Cincinnati Children’s Hospital Medical Center, Cincinnati, Ohio, USA

## Abstract

**Background:**

Polybrominated diphenyl ethers (PBDEs) reduce blood concentrations of thyroid hormones in laboratory animals, but it is unclear whether PBDEs disrupt thyroid hormones in pregnant women or newborn infants.

**Objectives:**

We investigated the relationship between maternal PBDE levels and thyroid hormone concentrations in maternal and cord sera.

**Methods:**

We used data from the Health Outcomes and Measures of the Environment (HOME)Study, a prospective birth cohort of 389 pregnant women in Cincinnati, Ohio, who were enrolled from 2003 through 2006 and delivered singleton infants. Maternal serum PBDE concentrations were measured at enrollment (16 ± 3 weeks of gestation). Thyroid hormone concentrations were measured in maternal serum at enrollment (*n* = 187) and in cord serum samples (*n* = 256).

**Results:**

Median maternal serum concentrations of BDEs 28 and 47 were 1.0 and 19.1 ng/g lipid, respectively. A 10-fold increase in BDEs 28 and 47 concentrations was associated with a 0.85-μg/dL [95% confidence interval (CI): 0.05, 1.64] and 0.82-μg/dL (95% CI: 0.12, 1.51) increase in maternal total thyroxine concentrations (TT_4_), respectively. Both congeners were also positively associated with maternal free thyroxine (FT_4_). We also observed positive associations between BDE-47 and maternal total and free triiodothyronine (TT_3_ and FT_3_). A 10-fold increase in BDE-28 was associated with elevated FT_3_ concentrations (β = 0.14 pg/mL; 95% CI: 0.02, 0.26). In contrast, maternal PBDE levels were not associated with thyroid hormone concentrations in cord serum.

**Conclusions:**

These findings suggest that maternal PBDE exposure, particularly BDEs 28 and 47, are associated with maternal concentrations of T_4_ and T_3_ during pregnancy.

**Citation:**

Vuong AM, Webster GM, Romano ME, Braun JM, Zoeller RT, Hoofnagle AN, Sjödin A, Yolton K, Lanphear BP, Chen A. 2015. Maternal polybrominated diphenyl ether (PBDE) exposure and thyroid hormones in maternal and cord sera: the HOME Study, Cincinnati, USA. Environ Health Perspect 123:1079–1085; http://dx.doi.org/10.1289/ehp.1408996

## Introduction

Polybrominated diphenyl ethers (PBDEs), synthetic flame retardants, have been used in the manufacture of consumer products, including furniture foam, carpet padding, and electronics. Because they are semivolatile and are not covalently bound to consumer products, PBDEs readily leach out into the environment. North Americans have the highest human concentrations of PBDEs globally, with serum levels 10–100 times higher than those observed among Europeans and Japanese (Costa and Giordano 2007).

PBDEs can reduce blood levels of thyroid hormones in laboratory animals (Darnerud 2008). Thyroxine (T_4_) was significantly reduced among rodents that were pre- and postnatally exposed to PBDEs (Kim TH et al. 2009a; Zhou et al. 2002), suggesting a hypothyroxinemic or hypothyroid effect. Possible mechanisms include competitive binding to the thyroid transport protein transthyretin (TTR) and thyroid hormone receptors (Meerts et al. 2000; Richardson et al. 2008), and increasing T_4_ metabolism and clearance by inducing thyroxine glucuronidation via uridine diphosphate glucuronosyltransferase enzymes (Zhou et al. 2002).

PBDEs may also interfere with adult human thyroid hormone levels, though studies suggest a hyperthyroid rather than a hypothyroid effect (Dallaire et al. 2009; Hagmar et al. 2001; Turyk et al. 2008). Because thyroid hormones are essential for fetal growth and neurological development, it is particularly important to test whether PBDEs alter thyroid hormone levels during pregnancy (Forhead and Fowden 2014). There is an increased demand on the maternal thyroid gland during pregnancy because the fetus relies predominantly on the maternal supply of thyroid hormones until approximately 18–22 weeks gestation (Morreale de Escobar et al. 2000). The fetus continues to depend on maternal inputs for thyroid hormone stabilization even after endogenous fetal production of thyroid hormones (Morreale de Escobar et al. 2004). Perturbations in thyroid hormone levels during gestation may result in altered neurobehavior. Lower levels of maternal T_4_ have been linked to neurodevelopmental deficits (Henrichs et al. 2013; Julvez et al. 2013), and maternal subclinical hypothyroidism has been associated with an increased risk of adverse pregnancy outcomes, including spontaneous abortion, placental abruption, and preterm delivery (Männistö et al. 2013).

Several epidemiologic studies have examined associations between PBDEs and thyroid hormone levels in maternal and cord sera (see Supplemental Material, Table S1). Though most studies have reported an association between PBDEs and one or more thyroid hormones, results are inconsistent (Abdelouahab et al. 2013; Chevrier et al. 2010; Herbstman et al. 2008; Kim TH et al. 2009b; Kim UJ et al. 2011; Lin et al. 2011; Mazdai et al. 2003; Roze et al. 2009; Stapleton et al. 2011; Zhang et al. 2010; Zota et al. 2011). Further, most have small to modest sample sizes, and only two have measured maternal thyroid hormones before full functioning of the fetal thyroid axis (Abdelouahab et al. 2013; Zhang et al. 2010).

Given the inconsistent results of studies linking PBDEs and thyroid hormones and the importance of thyroid hormones during pregnancy, we examined the relationships between prenatal PBDE exposure and thyroid hormone concentrations measured in maternal serum at 16 ± 3 weeks of gestation and in cord serum following delivery.

## Methods

*Study participants and design*. We used data from the Health Outcomes and Measures of the Environment (HOME) Study, an ongoing prospective pregnancy and birth cohort (http://www.cincinnatichildrens.org/research/divisions/e/environmental/study/default/). Nine prenatal clinics in the Cincinnati, Ohio (USA), metropolitan area served as the source population for pregnant women. Women were enrolled in the study between March 2003 and January 2006 if they were ≥ 18 years of age, residing in a house built before 1978 (a criterion relating to a goal of the larger HOME Study examining lead exposures), intending to continue prenatal care and deliver at any of the collaborating obstetric practices and hospitals, and HIV negative. Women receiving seizure, thyroid, or chemotherapy/radiation medications were ineligible to participate. Letters were mailed to women ≥ 18 years of age who were living in houses built before 1978 (*n* = 5,184). Of 1,263 eligible women, 468 enrolled and provided informed consent, and 389 remained in the study until they delivered live singleton infants. We further restricted the sample to women with PBDE concentrations measured at enrollment (16 ± 3 weeks of gestation) and thyroid hormone levels measured in either maternal (*n* = 187) or cord serum (*n* = 256). The study protocol was approved by the Institutional Review Board at the Cincinnati Children’s Hospital Medical Center and the Centers for Disease Control and Prevention (CDC). All participants provided written informed consent for themselves and their children before study enrollment.

*Data collection*. We collected sociodemographic, behavioral, and general health characteristics using standardized questionnaires and interviews administered following consent. Chart abstraction after delivery was used to obtain data on obstetric history and delivery.

Concentrations of PBDEs were measured in maternal serum samples collected at approximately 16 weeks gestation. Serum was separated from clotted blood samples and stored at –80°C until analysis. Concentrations of PBDEs (BDEs 17, 28, 47, 66, 85, 99, 100, 153, 154, and 183) and polychlorinated biphenyls (PCBs) were measured at the CDC using gas chromatography/isotope dilution high-resolution mass spectrometry (Jones et al. 2012; Sjödin et al. 2004). Serum samples were pretreated and extracted by solid phase extraction. Quality control (*n* = 3) and method blank (*n* = 3) samples were included in every batch of serum samples analyzed (*n* = 24). PBDE concentrations were expressed on a serum lipid basis (nanograms per gram). Total lipids were determined based on serum measurements of triglycerides and total cholesterol using standard enzymatic methods (Phillips et al. 1989). The limit of detection (LOD) was defined as three times the standard deviation (SD) of the method blank samples analyzed in parallel with the study samples or in the absence of a detectable blank as 5 pg/μL. Values < LOD were substituted with the LOD divided by the square root of 2 (Hornung and Reed 1990).

Thyroid hormone analysis was conducted by the Department of Laboratory Medicine of the University of Washington. Specimens were immediately stored at –70°C on arrival at the laboratory. Thyroid hormones and antibodies were quantified using an Access2 automated clinical immunoassay analyzer by Beckman Coulter, Inc. (Fullerton, CA). Two levels of quality control materials [BioRad Liquicheck or BioRad Immunoassay Plus (Hercules, CA)] were run with each assay every day (*n* = 22). Coefficients of variation for assays for thyroid-stimulating hormone (TSH), free thyroxine (FT_4_), total thyroxine (TT_4_), total triiodothyronine (TT_3_), free triiodothyronine (FT_3_), thyroid peroxidase (TPOAb), and thyroglobulin antibodies (TgAb) ranged from < 1.0 to 11% (see Supplemental Material, Table S2). To ensure against transcription errors, results were double-checked by a second technologist.

*Statistical analysis*. We computed summary statistics for individual and total PBDEs (ΣPBDEs), defined as the sum of congeners with detection frequencies > 50% (BDEs 28, 47, 99, 100, and 153). We evaluated correlations between PBDEs and thyroid hormones using Spearman rank-order correlation and analysis of variance to examine the relation between maternal and infant characteristics and ΣPBDE concentrations. The distribution of PBDEs, PCBs, TSH, and thyroid antibodies were log-normally distributed (Shapiro–Wilkes); therefore, concentrations of PBDEs and PCBs were log_10_-transformed, and TSH and thyroid antibodies were natural-log (ln) transformed.

We used separate multiple linear regression models to estimate β coefficients and 95% confidence intervals (CIs) for individual PBDE congeners with detection frequencies > 50% and ΣPBDEs in relation to each thyroid hormone in maternal and cord sera. Covariates included in final regression models were based on results of bivariate analyses examining the relationship with thyroid hormone levels (*p* < 0.20). Final maternal serum models included the following covariates (categorized as shown in Table 1): maternal age at enrollment, race, education, parity, family income, smoking status, alcohol consumption, gestational age at thyroid hormone measurement (in weeks, continuous), and maternal serum PCB concentrations (the sum of congeners with detection frequencies > 75%, including congeners 28, 74, 99, 105, 118, 146, 153, 156, 170, 180, 183, 187, 194, 199, and 206; log_10_-transformed, continuous). Cord serum models additionally included infant sex and mode of delivery. The following covariates were also considered, but did not meet our criteria for inclusion in the final models (*p* < 0.20): maternal blood lead levels, marijuana use, maternal country of birth, maternal depressive symptoms (Beck et al. 1996), vitamin intake (daily, < daily, never), and time of sample collection (hour of day). Percent changes in thyroid hormone concentrations associated with 10-fold increases in individual or ΣPBDEs were calculated by dividing the PBDE model coefficient by the mean thyroid hormone concentrations for the study sample (see Supplemental Material, Table S3).

**Table 1:** Serum concentrations of total PBDEs*^a^* (ng/g lipid) by demographic characteristics, HOME Study.

Characteristic	*n* (%)^*b*^	GM (GSD)
Age (years)
< 25	64 (23.3)	47.1 (2.2)*
25–34	165 (60.2)	41.2 (2.8)
≥ 35	45 (16.4)	28.4 (2.5)
Race/ethnicity
Non-Hispanic white	175 (64.3)	33.9 (2.6)**
Non-Hispanic black and others	97 (35.7)	53.7 (2.6)
Education
High school or less	71 (26.1)	56.4 (2.3)**
Some college or 2-year degree	65 (23.9)	42.6 (2.3)
Bachelor’s	84 (30.9)	34.1 (2.7)
Graduate or professional	52 (19.1)	29.7 (3.1)
Parity
Nulliparous	126 (46.0)	35.5 (2.7)
Primiparous	81 (29.6)	42.8 (2.5)
Multiparous	67 (24.4)	45.8 (2.7)
Mode of delivery
Vaginal	173 (63.1)	42.1 (2.5)
Planned cesarean	58 (21.1)	37.5 (3.1)
Emergency cesarean	18 (6.6)	26.1 (2.9)
Assisted vaginal	25 (9.1)	43.7 (2.1)
Breastfeeding current child
No	52 (19.6)	42.7 (2.2)
Yes	213 (80.4)	39.2 (2.7)
Breastfed previous child(ren)
No	36 (25.7)	53.1 (2.4)
Yes	104 (74.3)	40.7 (2.5)
Family income
< $40,000	106 (39.0)	52.0 (2.6)**
$40,000–$79,999	90 (33.1)	38.3 (2.6)
≥ $80,000	76 (27.9)	29.2 (2.5)
Smoking status
No	225 (82.1)	38.0 (2.7)
Environmental tobacco smoke	26 (9.5)	43.1 (2.2)
Active	23 (8.4)	59.2 (2.4)
Alcohol consumption
Never	154 (56.6)	41.4 (2.6)
< 1 Alcoholic drink per month	82 (30.1)	37.1 (2.7)
> 1 Alcoholic drink per month	36 (13.2)	40.7 (2.8)
Marijuana use
No	251 (92.3)	39.2 (2.7)
Yes	21 (7.7)	50.3 (2.4)
Infant sex
Male	122 (44.5)	37.0 (2.6)
Female	152 (55.5)	42.5 (2.7)
Birth weight (g)
< 2,500	13 (4.7)	51.1 (2.7)
2,500–3,500	145 (52.9)	41.1 (2.5)
> 3,500	116 (42.3)	37.5 (2.8)
Abbreviations: GM, geometric mean; GSD, geometric standard deviation. ^***a***^Sum of congeners with detection frequencies > 50% (BDEs 28, 47, 99, 100, and 153). ^***b***^Frequencies may not add to the total number of participants because of missing values. Percentages may not add to 100% because of rounding. *p < 0.05. **p < 0.001 (two-sided *p*-values using analysis of variance).		

We estimated dose–response models by linear regression for individual PBDE congeners using quartiles, with quartile 1 as the referent group. Linear trend was assessed by using the median value in each quartile as a continuous variable in the linear regression models (Greenland 1995). We also examined the relation between prenatal PBDE exposure and thyroid antibody concentrations (TgAb or TPOAb) in maternal and cord sera using linear regression models. Because women or infants with impaired thyroid function may be more susceptible to the effects of PBDE exposure, we examined whether thyroid antibodies modified the association between PBDEs and thyroid hormones using product interaction terms between continuous PBDE concentrations and dichotomous TgAb or TPOAb (*p* < 0.10 considered significant). Few participants had clinically significant levels of TgAb (> 2.0 IU/mL; *n* = 8), modified from previous laboratory reference range [National Health and Nutrition Examination Survey (NHANES) 2007a], or TPOAb (> 9.0 IU/mL; *n* = 15) (NHANES 2007b). Therefore, we dichotomized TPOAb at > or ≤ the median level and TgAb at detectable or not detectable. Stata version 12.1 (StataCorp, College Station, TX) was used for statistical analyses, and graphs were produced using GraphPad Prism (GraphPad, San Diego, CA). All tests of statistical significance were two-sided, and *p* < 0.05 were considered significant.

## Results

BDEs 28, 47, 99, 100, and 153, major components of the penta mixture DE-71, had detection frequencies ranging from 80% to 100% (Table 2). The most abundant congener was BDE-47, with a geometric mean (GM) of 20.5 ng/g lipid. Concentrations of ΣPBDEs were higher among women who were younger, less educated, and of lower income (Table 1). Further, women who self-reported as non-Hispanic white had lower concentrations of ΣPBDEs (33.9 ± 2.6 ng/g lipid) compared with non-Hispanic blacks and others (53.7 ± 2.6 ng/g lipid). Although not statistically significant, concentrations of ΣPBDEs were higher among active smokers and women whose infants were < 2,500 g. As expected, PBDE congeners were highly correlated with each other and with ΣPBDEs (*r*_S_ = 0.47–0.96, *p* < 0.0001) (see Supplemental Material, Table S4).

**Table 2:** Concentrations of PBDE congeners (ng/g lipid) around the 16th week of pregnancy, HOME Study.

PBDEs	*n*	Percent detection	Minimum	Percentile				Maximum	GM (GSD)	NHANES^*a*^ GM (GSD)
				25th	50th	75th	95th			
∑PBDEs^*b*^^,^^*c*^	274	100.0^*d*^	4.5	20.8	36.0	75.1	213.7	2046.9	40.0 (2.6)	NA
BDE-17	275	3.3	0.1	0.3	0.3	0.4	1.0	4.0	NA	NA
BDE-28^*c*^	275	80.0	0.2	0.6	1.0	1.7	4.8	31.4	1.1 (2.4)	1.5 (0.3)^*e*^
BDE-47^*c*^	305	100.0	1.5	10.8	19.1	35.3	103.0	1,290	20.5 (2.7)	23.9 (2.2)
BDE-66	275	1.8	0.1	0.3	0.3	0.4	1.1	2.6	NA	NA
BDE-85	275	49.5	0.2	0.3	0.5	1.0	3.7	38.7	NA	NA
BDE-99^*c*^	294	99.3	0.6	2.5	4.4	8.8	32.8	465	4.9 (2.9)	5.5 (0.8)
BDE-100^*c*^	275	98.2	0.4	2.1	3.7	7.9	27.6	172	4.1 (2.9)	6.1 (0.9)
BDE-153^*c*^	274	99.3	0.5	2.7	4.5	9.0	50.8	152	5.5 (2.9)	9.9 (3.0)
BDE-154	275	44.7	0.2	0.3	0.5	1.0	3.0	28.7	NA	NA
BDE-183	275	22.9	0.1	0.3	0.4	0.5	1.2	9.3	NA	NA
Abbreviations: GM, geometric mean; GSD, geometric standard deviation; NA, not available (percent detection < 50%). ^***a***^Serum concentrations in NHANES (National Health and Nutrition Examination Survey) 2003–2004 pregnant women (Woodruff et al. 2011). ^***b***^Includes congeners with detection frequencies > 50% (BDEs 28, 47, 99, 100, and 153). ^***c***^Included in statistical analyses. ^***d***^Percentage of samples with at least one congener above the LOD. ^***e***^Arithmetic mean concentration among NHANES 2007–2008 individuals ages 20–39 years (Sjödin et al. 2014).										

The GM of TSH was higher in cord serum (7.1 ± 1.8 μIU/mL) than in maternal serum (1.2 ± 2.2 μIU/mL) (see Supplemental Material, Table S3). Cord TT_3_ levels (51.5 ± 18.9 ng/dL) were approximately a third of maternal levels (160.5 ± 24.1 ng/dL). Maternal TSH was weakly positively correlated with cord TSH (*r*_S_ = 0.26; *p* < 0.01) (see Supplemental Material, Table S5). Positive correlations were observed between TT_4_ and FT_4_, TT_3_, and FT_3_ in maternal and cord sera.

Individual PBDE congeners and ΣPBDEs were inversely related to maternal serum concentrations of TSH; however, none of the associations were statistically significant (Table 3). We estimated significant increases in maternal TT_4_ for each 10-fold increase of BDE-28 (β = 0.85 μg/dL; 95% CI: 0.05, 1.64) and BDE-47 (β = 0.82 μg/dL; 95% CI: 0.12, 1.51), corresponding to an 8% (95% CI: 0.5%, 16%) and 8% (95% CI: 1%, 15%) increase in mean maternal TT_4_, respectively. We also found that 10-fold increases in BDEs 28 and 47 were associated with maternal FT_4_ increases of 7% (95% CI: 1%, 13%) and 6% (95% CI: 1%, 10%), respectively. Ten-fold increases in BDEs 28 and 47 were also associated with higher concentrations of maternal FT_3_ (β = 0.14 pg/mL; 95% CI: 0.02, 0.26 and β = 0.12 pg/mL; 95% CI: 0.01, 0.22), which were equivalent to increases of 4% (95% CI: 1%, 8%) and 4% (95% CI: 0.3%, 7%) from the mean FT_3_, respectively. BDE-47 was significantly associated with maternal serum TT_3_ (β = 8.71 ng/dL; 95% CI: 0.42, 16.99), corresponding to an increase of 5% (95% CI: 0.3%, 11%) from the mean maternal TT_3_ level of 160.5 ng/dL. Concentrations of individual PBDE congeners were generally associated with lower concentrations of cord TSH, T_4_, and T_3_; however, only one significant inverse association was observed between BDE-28 and FT_3_, with a 6% (95% CI: –12%, –0.2%) decrease from the mean concentration of cord FT_3_ for every 10-fold increase in BDE-28.

**Table 3:** Adjusted associations between maternal PBDE concentrations and maternal and cord sera levels of thyroid hormones, HOME Study.*^a^*

PBDEs	Maternal serum^*b*^		Cord serum^*b*^^,^^*c*^	
	*n*	β (95% CI)	*n*	β (95% CI)
lnTSH
BDE-28	165	–0.07 (–0.40, 0.26)	228	–0.04 (–0.24, 0.16)
BDE-47	165	–0.14 (–0.43, 0.15)	228	–0.10 (–0.28, 0.07)
BDE-99	165	–0.12 (–0.40, 0.16)	228	–0.16 (–0.33, 0.01)
BDE-100	165	–0.09 (–0.37, 0.18)	228	–0.04 (–0.21, 0.12)
BDE-153	165	–0.10 (–0.35, 0.16)	227	0.06 (–0.10, 0.22)
∑PBDEs	165	–0.11 (–0.41, 0.19)	227	–0.08 (–0.26, 0.10)
TT_4_
BDE-28	165	0.85 (0.05, 1.64)*	224	–0.13 (–0.78, 0.52)
BDE-47	165	0.82 (0.12, 1.51)*	224	–0.15 (–0.72, 0.43)
BDE-99	165	0.49 (–0.19, 1.16)	224	–0.08 (–0.63, 0.48)
BDE-100	165	0.55 (–0.12, 1.22)	224	–0.15 (–0.69, 0.39)
BDE-153	165	0.07 (–0.56, 0.70)	223	–0.08 (–0.60, 0.45)
∑PBDEs	165	0.61 (–0.11, 1.33)	223	–0.15 (–0.74, 0.44)
TT_3_
BDE-28	165	9.34 (–0.19, 18.88)	228	–4.34 (–10.76, 2.07)
BDE-47	165	8.71 (0.42, 16.99)*	228	–1.71 (–7.39, 3.97)
BDE-99	165	6.30 (–1.70, 14.30)	228	–0.47 (–5.97, 5.04)
BDE-100	165	7.04 (–0.93, 15.01)	228	–0.58 (–5.94, 4.77)
BDE-153	165	1.15 (–6.35, 8.65)	227	2.03 (–3.17, 7.23)
∑PBDEs	165	6.38 (–2.23, 14.99)	227	–0.47 (–6.32, 5.38)
FT_4_
BDE-28	165	0.05 (0.01, 0.09)*	228	–0.04 (–0.09, 0.02)
BDE-47	165	0.04 (0.004, 0.07)*	228	–0.03 (–0.07, 0.02)
BDE-99	165	0.02 (–0.01, 0.05)	228	–0.01 (–0.05, 0.03)
BDE-100	165	0.02 (–0.01, 0.05)	228	–0.03 (–0.07, 0.02)
BDE-153	165	0.004 (–0.03, 0.04)	227	–0.03 (–0.07, 0.02)
∑PBDEs	165	0.03 (–0.01, 0.06)	227	–0.04 (–0.08, 0.01)
FT_3_
BDE-28	165	0.14 (0.02, 0.26)*	226	–0.11 (–0.21, –0.003)*
BDE-47	165	0.12 (0.01, 0.22)*	226	–0.06 (–0.15, 0.03)
BDE-99	165	0.07 (–0.03, 0.17)	226	–0.03 (–0.12, 0.06)
BDE-100	165	0.08 (–0.02, 0.18)	226	–0.04 (–0.13, 0.05)
BDE-153	165	0.001 (–0.09, 0.10)	225	–0.01 (–0.09, 0.08)
∑PBDEs	165	0.10 (–0.01, 0.21)	225	–0.05 (–0.14, 0.04)
^***a***^Units: PBDEs (ng/g lipid), TSH (μIU/mL), TT_4_ (μg/dL), TT_3_ and FT_4_ (ng/dL), and FT_3_ (pg/mL). PBDEs were log_10_-­transformed. ^***b***^Adjusted for maternal age, race/ethnicity, education, parity, family income, smoking status, alcohol consumption, gestational age at blood draw, and total serum PCB concentrations. ^***c***^Additionally adjusted for infant sex and mode of delivery. *p < 0.05.				

**Figure 1:**
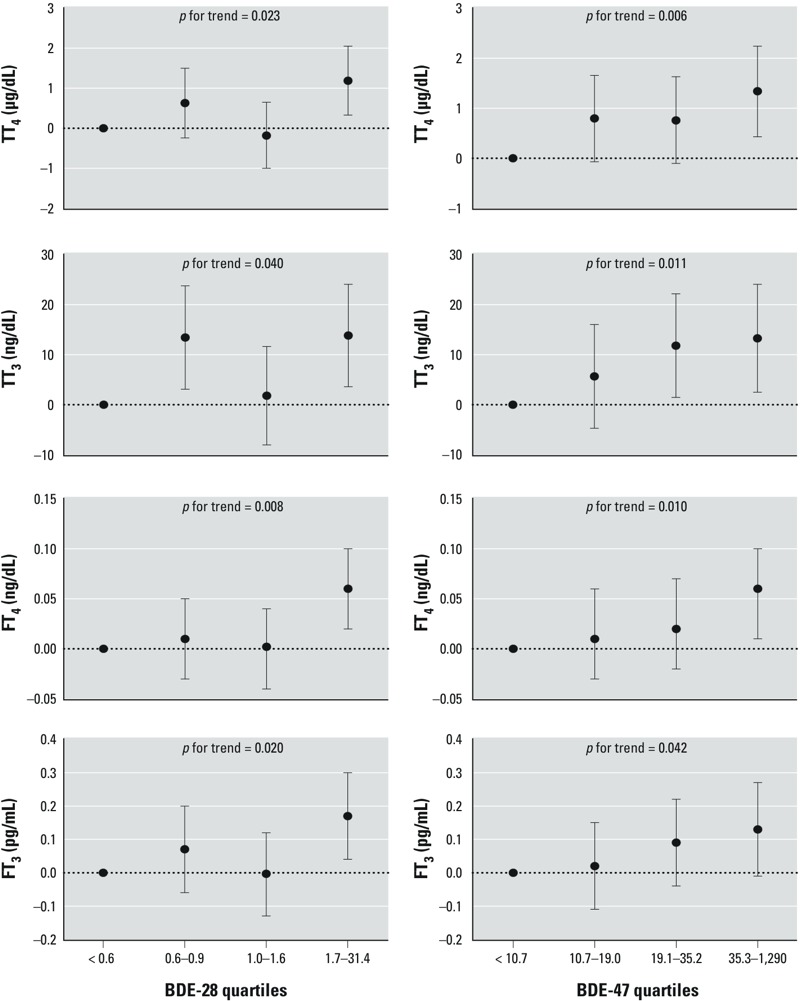
β-coefficients and 95% CIs from regression models for associations of BDE-28 and BDE-47 (ng/g lipid) quartiles and maternal thyroid hormones. All models adjusted for maternal age, race/ethnicity, education, parity, family income, smoking status, alcohol consumption, gestational age at serum collection, and total serum PCB concentrations. *p*-Value for trend was obtained by using the median value in each quartile as a continuous variable in the linear regression models.

We observed a significant linear trend between quartiles of BDE-47 and concentrations of maternal free and total T_4_ and T_3_, particularly with TT_4_ (*p* trend = 0.006) (Figure 1). Significant linear trend was also observed with BDE-28 and maternal free and total T_4_ and T_3_; however, the pattern may suggest a nonmonotonic relationship. No significant linear trend was noted between BDE-28 or BDE-47 quartiles and thyroid hormones in cord serum (see Supplemental Material, Figure S1).

No significant relationship was detected between PBDEs and thyroid antibody concentrations in maternal or cord serum (see Supplemental Material, Table S6). However, TgAb significantly modified (*p* < 0.10) the association between PBDEs (BDEs 47 and 99, and ΣPBDEs) and cord TSH and FT_3_ (see Supplemental Material, Table S7). PBDEs were negatively associated with cord TSH and FT_3_ among women with detectable TgAb, and associations were null for those with undetectable concentrations. We also observed that ΣPBDEs were associated with reductions in cord FT_4_ concentrations if cord TPOAb was above the median (0.3 IU/mL), which may be attributable to a lower capacity of T_4_ production, and subsequently lower transfer of T_4_ to the fetus, in women with high TPOAb.

## Discussion

We found that maternal concentrations of BDEs 28 and 47 during pregnancy were associated with increased concentrations of maternal free and total T_4_ and T_3_ in the early second trimester of pregnancy. A 4–8% increase in maternal T_3_ and T_4_ levels from the study sample mean values was observed with 10-fold increases in BDEs 28 and 47. This association may be attributable to structural similarities between these congeners and T_3_ and T_4_. BDEs 28 and 47, which have three and four bromines on their two phenyl rings, more closely resemble T_3_ and T_4_, which contain three and four iodine atoms, respectively.

Although we estimated increases in mean T_3_ and T_4_ that are below clinical thresholds, the difference may not be trivial; small shifts in the distribution may have a substantial impact (Miller et al. 2009). In a longitudinal study of 16 healthy men, Andersen et al. (2002) obtained monthly serum samples for 1 year and observed that the width of the individual 95% CIs was approximately half that of the group for free and total T_4_, T_3_, and TSH, suggesting that small shifts at a population level would reflect relatively large changes on an individual level. Further, the importance of this elevation is unclear, especially among subpopulations that may be more sensitive to thyroid hormone disruption, such as individuals with clinical or subclinical hyperthyroxinemia.

Our findings regarding TT_4_ and FT_4_ are similar to those reported by Stapleton et al. (2011), in which increases in ln-BDE-47 were associated with higher maternal levels of TT_4_ (β = 0.42 μg/dL; 95% CI: 0.05, 0.78) and FT_4_ (β = 0.05 ng/dL; 95% CI: 0.01, 0.08) during the third trimester. We observed findings similar to those of Stapleton et al. (2011) regarding positive associations between maternal TT_4_ and BDEs 99 and 100, and FT_4_ and BDE-153, but associations were not statistically significant (Table 3). A recent study of 260 Canadian women measured PBDE concentrations and thyroid hormones at 10.8 ± 2.7 weeks of gestation and reported similar results regarding PBDEs and FT_4_, finding that BDE-47 (β = 0.02 ng/dL; 95% CI: 0.005, 0.03) and BDE-99 (β = 0.02 ng/dL; 95% CI: 0.005, 0.04) were associated with increased concentrations of FT_4_ during the first trimester (Abdelouahab et al. 2013). In contrast to our findings and those of Stapleton et al. (2011), an inverse association was noted between PBDEs and maternal TT_4_ in the Canadian study. Further, studies examining the role of PBDEs on thyroid hormone levels in men have likewise reported positive associations with TT_4_ and FT_4_ (Meeker et al. 2009; Turyk et al. 2008). A positive association between PBDEs and maternal TT_3_ was also reported by Stapleton et al. (2011), in which BDE-47 was associated with maternal TT_3_ levels > 178 ng/dL (odds ratio = 1.30; 95% CI: 1.00, 1.69). However, an inverse association was estimated with serum concentrations of BDE-47 (β = –7.81 ng/dL; 95% CI: –11.37, –4.26) and BDE-99 (β = –4.19 ng/dL; 95% CI: –8.26, –0.12) and maternal TT_3_ by Abdelouahab et al. (2013), though an increase in maternal FT_3_ with prenatal BDE-99 exposure was also reported (β = 0.08 pmol/L; 95% CI: 0.03, 0.13).

Consistent with other studies, we found no significant relation between PBDEs and TSH in maternal (Abdelouahab et al. 2013; Stapleton et al. 2011; Zhang et al. 2010) and cord sera (Abdelouahab et al. 2013; Herbstman et al. 2008; Kim TH et al. 2009b; Lin et al. 2011; Roze et al. 2009). In addition, our results are consistent with previous studies reporting no association between PBDEs and cord levels of free and total T_4_ (Kim TH et al. 2009b; Lin et al. 2011; Mazdai et al. 2003; Roze et al. 2009). Cord serum analyses yielded one significant result between BDE-28 and FT_3_. Only one previous study, conducted in a sample of 54 pregnant women in southern Taiwan, reported a reduction in FT_3_ levels in cord serum with BDEs 153 and 183 exposure (Lin et al. 2011). Although not statistically significant, prenatal PBDEs consistently had an inverse relationship with free and total T_4_ and T_3_ in cord serum in our study. This was unexpected given the increases observed in maternal thyroid hormones. However, inconsistent results between prenatal PBDEs and thyroid hormones in maternal and cord sera have also been reported by Abdelouahab et al. (2013). It is not certain what mechanisms would result in these contrasting findings. It is noteworthy that previous studies have focused on different gestational periods, and thyroid hormones are known to fluctuate during pregnancy (Soldin et al. 2004). Inconsistent conclusions between studies may be attributable to differences in timing of thyroid hormone measurements—an issue we attempted to address by controlling for gestational age at blood draw and using an exposure–outcome measure within a relatively narrow time window (16 ± 3 weeks of gestation). Differences in the exposure levels across populations could also explain discrepancies.

Most rodent models have shown a reduction in serum T_4_ concentrations with PBDE exposure, suggesting a hypothyroxinemic effect (Richardson et al. 2008; Zhou et al. 2002), whereas human studies in nonpregnant cohorts suggest a hyperthyroid effect (Hagmar et al. 2001; Meeker et al. 2009; Turyk et al. 2008). Conflicting reports between animal and human studies may stem from physiological differences across species. Although hydroxylated-PBDEs (OH-PBDEs) were not measured in our study, these metabolites bind to TTR with high potency. However, the percentage of TTR-bound T_4_ is comparatively lower in humans than in rodents because the major thyroid hormone transport protein in humans is thyroxine-binding globulin (TBG). The mechanisms by which PBDEs affect thyroid hormone action may be further complicated by the potential effects of various metabolites.

Most studies examining PBDEs and thyroid hormone levels have assessed either pregnant women during late gestation or fetal levels of thyroid hormones in cord serum. To our knowledge, only one other study has examined PBDEs and thyroid hormones during the second trimester of pregnancy. However, this study had a rather small sample size of 25 pregnant women between 19 and 23 weeks of gestation (Zota et al. 2011). Chevrier et al.’s (2010) study population comprised 270 pregnant women, but PBDEs were measured at 27.3 ± 3.1 weeks of gestation, the last week of the second trimester. Both studies reported null associations between prenatal PBDEs and free and total T_4_, whereas positive associations were observed in our study. In addition, although our findings do not indicate a relation between PBDEs and maternal or cord TSH, Chevrier et al. (2010) reported a decrease in maternal TSH with 10-fold increases in BDE-100 exposure (β = –0.09 mIU/L; 95% CI: –0.15, –0.02), and Zota et al. (2011) reported an increase with ln-BDE-85 (β = 0.33 mIU/L; 95% CI: 0.02, 0.64).

Our study has several strengths. We included data on numerous potentially confounding covariates, encompassing sociodemographic and behavioral factors, mode of delivery, time of sample collection, and PCB concentrations. Further, PBDE concentrations in our participants are comparable to pregnant women from NHANES during 2003–2004. The GM of BDE-47 in the NHANES pregnant women was 23.9 ng/g lipid (Woodruff et al. 2011) compared with 20.5 ng/g lipid observed in our study. The arithmetic mean concentration of BDE-28 among individuals 20–39 years of age in NHANES 2007–2008 (1.5 ng/g lipid) (Sjödin et al. 2014) was also similar to that of our participants (1.8 ng/g lipid). In addition, thyroid hormone levels in the majority of our study participants are within the normal range for pregnant women (Soldin et al. 2004) and are similar to those of other study populations (Abdelouahab et al. 2013; Stapleton et al. 2011; Zota et al. 2011).

We examined interactions between PBDEs and thyroid antibodies. Ten-fold increases in certain PBDEs were associated with reductions in cord TSH and FT_3_ if cord TgAb levels were detectable. Lower concentrations of cord FT_4_ were observed with exposure to PBDEs at TPOAb levels above the median, which is biologically plausible given that women with high levels of TPOAb have a lower capacity for T_4_ production and thus lower transfer of T_4_ to the fetus.

Our findings are subject to several limitations. First, we were unable to examine OH-PBDEs, which may be more detrimental to the thyroid system than their parent congeners because OH-PBDEs more closely resemble T_3_ and T_4_, have a higher affinity to TBG and TTR (Marchesini et al. 2008; Meerts et al. 2000), and may increase deiodination of T_4_ (Stapleton et al. 2009). Urinary iodine was measured at 26 weeks gestation in a majority of study participants, which may not reflect the iodine levels at the time of thyroid hormone measurements (16 weeks gestation or at delivery) because iodine has a short half-life and varies substantially daily (König et al. 2011). Therefore, we did not include iodine as a covariate.

Competitive binding may be an issue when using immunoassays to measure thyroid hormones. Antibodies used to bind with thyroid hormones may also be able to bind with PBDEs because they are structurally similar, making PBDEs appear positively correlated with thyroid hormones. However, a positive association has been observed between PBDEs and T_3_ levels in placental tissue measured by liquid chromatography-tandem mass spectrometry (Leonetti et al. 2014). Last, free thyroid hormones were measured using an immunoassay that may be subject to measurement error. Bound-T_4_ (TBG-attached T_4_) increases during pregnancy and may affect immunoassay results (Wang et al. 2000). Equilibrium dialysis may be more appropriate because this method has been shown to yield accurate results regardless of elevated bound-T_4_ concentrations (Nelson et al. 1994). Aside from Chevrier et al. (2010), no researchers examining prenatal PBDEs and thyroid hormones in maternal or cord serum have performed equilibrium dialysis. Limited volume of residual serum samples precluded us from further pursuing this method.

## Conclusions

We observed that levels of maternal serum BDEs 28 and 47 were associated with increases in maternal serum concentrations of T_4_ and T_3_ during the early second trimester of pregnancy. However, the changes in thyroid concentrations were in the subclinical range, and their potential impact is unclear. In contrast, we did not find that prenatal PBDE exposure was related to thyroid hormone concentrations in cord serum, nor was there evidence to suggest a relation between PBDEs and thyroid hormone antibodies in maternal or cord serum. Future studies should focus on OH-PBDEs and thyroid hormones during early gestation. Additional research is also needed to explore mechanisms by which PBDEs and their metabolites exert their action on the thyroid system and to identify susceptible populations.

## Supplemental Material

(605 KB) PDF
