# Supplementary material for: Maternal Polybrominated Diphenyl Ether (PBDE) Exposure and Thyroid Hormones in Maternal and Cord Sera: The HOME Study, Cincinnati, USA
Source: Environ Health Perspect. 2015 Apr 17;123(10):1079–85. doi: 10.1289/ehp.1408996 (PMC4590759; doi:10.1289/ehp.1408996)
Supplement: (605 KB) PDF [file ehp.1408996.s001.acco.pdf]

Environ Health Perspect
doi: 10.1289/ehp.1408996

Note to Readers: *EHP* strives to ensure that all journal content is accessible to all readers. However, some figures and Supplemental Material published in *EHP* articles may not conform to 508 standards due to the complexity of the information being presented. If you need assistance accessing journal content, please contact ehp508@niehs.nih.gov. Our staff will work with you to assess and meet your accessibility needs within 3 working days.

Supplemental Material

Maternal Polybrominated Diphenyl Ether (PBDE) Exposure and Thyroid Hormones in Maternal and Cord Sera: The HOME Study, Cincinnati, USA

Ann M. Vuong, Glenys M. Webster, Megan E. Romano, Joseph M. Braun, R. Thomas Zoeller, Andrew N. Hoofnagle, Andreas Sjödin, Kimberly Yolton, Bruce P. Lanphear, and Aimin Chen

Table of Contents

Table S1. Comparison of published studies on polybrominated diphenyl ether and thyroid hormone levels in pregnancy.

Table S2. Coefficients of variation for thyroid hormone and antibody assays.

Table S3. Thyroid hormone levels, HOME Study.

Table S4. Spearman rank correlation matrix for polybrominated diphenyl ether congeners and thyroid hormones, HOME Study.

Table S5. Spearman rank correlation matrix for thyroid hormone levels in maternal and cord sera, HOME Study.

Table S6. Adjusted associations between maternal polybrominated diphenyl ether concentrations and maternal and cord sera levels of thyroid antibodies, HOME Study.

Table S7. Associations between polybrominated diphenyl ether concentrations and select thyroid hormones in maternal and cord sera by thyroid antibody levels, HOME Study.

Figure S1. β-coefficients and 95% CIs from regression models for associations of BDE-28 and BDE-47 (ng/g lipid) quartiles and thyroid hormones in cord serum. All models adjusted for

maternal age, race/ethnicity, education, parity, family income, smoking status, alcohol consumption, infant gender, mode of delivery, gestational age at serum collection, and total serum PCB concentrations. *P* trend was obtained by using the median value in each quartile as a continuous variable in the linear regression models.

References

Table S1. Comparison of published studies on polybrominated diphenyl ether and thyroid hormone levels in pregnancy.[a]

First Author, Year	Study Size	Time of Sampling (weeks of gestation)		PBDEs	TSH	TT_4	TT_3	FT_4	FT_3
		PBDEs	THs						
Abdelouahab et al., 2013[b]	380	10±3	10±3	BDE-47<br>BDE-99<br>BDE-100<br>BDE-153	NS	↓ (∑PBDEs, BDE-47, -99)	↓ (∑PBDEs, BDE-47, -99)	↑ (∑PBDEs, BDE-47, -99)	↑ (∑PBDEs, BDE-99)
	260	10±3	Delivery		NS	↓ (BDE-99)	↓ (∑PBDEs, BDE-47)[c]	↓ (BDE-99)[c]	↓ (∑PBDEs, BDE-47)
	260	10±3	Cord Serum		NS	↓ (∑PBDEs, BDE-47, -99)	NS	↓ (∑PBDEs, BDE-47, -99)	–
Vuong et al., (current study)[d]	187	16±3	16±3	BDE-28<br>BDE-47<br>BDE-99<br>BDE-100<br>BDE-153	NS	↑ (BDE-28, -47)	↑ (BDE-47)	↑ (BDE-28, -47)	↑ (BDE-28, -47)
	256	16±3	Cord Serum		NS	NS	NS	NS	↓ (BDE-28)
Zota et al., 2011[b]	25	19-23	19-23	BDE-28<br>BDE-47<br>BDE-85<br>BDE-99<br>BDE-100<br>BDE-153<br>BDE-207	↑ (BDE-85)<br>↓ (BDE-207)	NS	–	NS	–
Chevrier et al., 2010[d]	270	27.3±3.1	27.3±3.1	BDE-28<br>BDE-47<br>BDE-99<br>BDE-100<br>BDE-153	↓	NS	–	NS	–
Stapleton et al., 2011[d]	136	>34	>34	BDE-47<br>BDE-99<br>BDE-100<br>BDE-153	NS	↑(∑PBDEs, BDE-47, -99, -100)	↑(BDE-47)	↑ (∑PBDEs, BDE-47, -153)	NS
Roze et al., 2009[e]	51	35	Cord Serum	BDE-47<br>BDE-99<br>BDE-100<br>BDE-153	NS	NS	↑(BDE-47, -99, -100)	NS	–

First Author, Year	Study Size	Time of Sampling (weeks of gestation)		PBDEs	TSH	TT_4	TT_3	FT_4	FT_3
		PBDEs	THs						
Mazdai et al., 2003[b]	9	Delivery	Delivery	BDE-47<br>BDE-99<br>BDE-100<br>BDE-153	−	NS	NS	NS	NS
	9	Cord Serum	Cord Serum		−	NS	NS	NS	NS
Herbstman et al., 2008[d]	289	Cord Serum	Cord Serum	BDE-47<br>BDE-100<br>BDE-153	NS	↓ (BDE-100)[f]	−	NS	−
Kim et al., 2009[g]	108	Cord Serum	Cord Serum	BDE-28<br>BDE-47<br>BDE-99<br>BDE-100	NS	−	−	NS	−
Zhang et al., 2010[g]	50	Cord Serum	16	∑PBDEs	NS	NS	NS	−	−
Kim et al., 2011[e]	90	Cord Serum	Cord Serum	BDE-28<br>BDE-47<br>BDE-99<br>BDE-100<br>BDE-153<br>BDE-154	NS	−	NS	↓ (BDE-28)	−
Lin et al., 2011[e]	54	Cord Serum	Cord Serum	BDE-15<br>BDE-28<br>BDE-47<br>BDE-99<br>BDE-100<br>BDE-153<br>BDE-154<br>BDE-184	NS	NS	↓ (BDE-154)	NS	↓ (BDE-153, -183)

Abbreviations: NS, not significant.

[a]↑ represents an increase; ↓ represents a decrease; − represents not studied. [b]BDE-47 median >20 ng/g lipid. [c]For volume-based models. [d]BDE-47 median 10-20 ng/g lipid. [e]BDE-47 median <10 ng/g lipid. [f]For infants delivered by spontaneous unassisted vaginal delivery. [g]BDE-47 median not provided.

Table S2. Coefficients of variation for thyroid hormone and antibody assays.

Thyroid hormone or antibody	Concentration	Coefficient of Variation (%)
TSH (uIU/mL)	0.7	5.8
	29.1	5.2
TT_4 (µg/dL)	5.1	6.9
	19.3	4.7
TT_3 (ng/dL)	83.7	7.3
	270.6	3.6
FT_4 (ng/dL)	0.6	<1.0
	4.8	5.9
FT_3 (pg/mL)	2.1	10.0
	8.9	6.1
TPOAb (IU/mL)	6.1	11.0
	56.1	9.3
TgAb (IU/mL)	4.6	9.6
	144.6	6.3

Table S3. Thyroid hormone levels, HOME Study.[a]

**Thyroid Hormones and Antibodies**	**Maternal Serum at 16 Weeks (n=187) Mean±SD or N (%)**	**Cord Serum (n=256)**
TSH[b]	1.2±2.2	7.1±1.8
TT_4	10.3±1.9	9.6±1.8
TT_3	160.5±24.1	51.5±18.9
FT_4	0.7±0.1	1.0±0.2
FT_3	3.2±0.3	1.7±0.3
TPOAb		
Above the median[c]	85(45.7)	118(46.6)
TgAb		
Detectable	66(35.5)	105(41.3)

Abbreviations: SD, standard deviation.

[a]TSH (uIU/mL), TT_4 (µg/dL), TT_3 and FT_4 (ng/dL), FT_3 (pg/mL), TPOAb and TgAb (IU/mL). [b]Geometric mean±SD. [c]Maternal serum: 0.6 IU/mL. Cord serum: 0.3 IU/mL.

Table S4. Spearman rank correlation matrix for polybrominated diphenyl ether congeners and thyroid hormones, HOME Study.[a]

Variable	BDE-28	BDE-47	BDE-99	BDE-100	BDE-153	∑PBDEs
PBDEs[b]						
BDE-28						
BDE-47	0.87##					
BDE-99	0.76##	0.92##				
BDE-100	0.78##	0.90##	0.85##			
BDE-153	0.50##	0.50##	0.47##	0.74##		
∑PBDEs	0.83##	0.92##	0.88##	0.96##	0.73##	
Thyroid hormones[c]						
Maternal Serum						
TSH	-0.04	-0.09	-0.10	-0.02	-0.04	-0.05
TT_4	0.16*	0.19*	0.18*	0.12	-0.02	0.13
TT_3	0.16*	0.25#	0.23**	0.21**	0.03	0.19*
FT_4	0.18*	0.17*	0.10	0.10	0.02	0.12
FT_3	0.18*	0.26#	0.18*	0.15	-0.01	0.18*
Cord Serum						
TSH	-0.07	-0.13*	-0.20**	-0.09	0.03	-0.13
TT_4	-0.02	-0.02	-0.01	-0.02	0.06	-0.02
TT_3	-0.12	-0.10	-0.08	-0.08	0.03	-0.08
FT_4	-0.05	-0.01	0.02	-0.01	-0.001	-0.03
FT_3	-0.12	-0.08	-0.06	-0.07	0.01	-0.08

*$p<0.05$, **$p<0.01$, #$p<0.001$, ##$p<0.0001$.

[a]Units: PBDE (ng/g lipid), TSH (uIU/mL), TT_4 (µg/dL), TT_3 and FT_4 (ng/dL), and FT_3 (pg/mL). [b]Total n for each of the pairwise correlations with BDE-28, -47, -99, -100, -153, and ∑PBDEs ranged from 274 to 294. [c]Total n for each of the pairwise correlations with thyroid hormones ranged from 166 to 187 for maternal serum and between 224 and 255 for cord serum.

Table S5. Spearman rank correlation matrix for thyroid hormone levels in maternal and cord sera, HOME Study.[a]

Thyroid Hormones	Maternal Serum					Cord Serum				
	**TSH**	**TT_4**	**TT_3**	**FT_4**	**FT_3**	**TSH**	**TT_4**	**TT_3**	**FT_4**	**FT_3**
Maternal Serum										
TSH										
TT_4	-0.16*									
TT_3	0.04	0.49**								
FT_4	-0.19**	0.48**	0.08							
FT_3	0.07	0.34**	0.69**	0.20**						
Cord Serum										
TSH	0.26**	-0.12	0.10	-0.13	0.06					
TT_4	0.04	0.02	0.06	-0.09	-0.06	-0.06				
TT_3	0.004	-0.09	0.11	-0.17*	0.07	0.12	0.38**			
FT_4	-0.01	-0.02	0.09	-0.02	-0.03	-0.10	0.58**	0.19**		
FT_3	-0.04	0.004	0.11	-0.11	0.06	0.09	0.31**	0.74**	0.23**	

*$p<0.05$, **$p<0.01$

[a]Units: TSH (uIU/mL), TT_4 (µg/dL), TT_3 and FT_4 (ng/dL), and FT_3 (pg/mL).

Table S6. Adjusted associations between maternal polybrominated diphenyl ether concentrations and maternal and cord sera levels of thyroid antibodies, HOME Study.[a]

Thyroid Antibodies	Maternal Serum[b] β (95% CI)	Cord Serum[b,c] β (95% CI)
TPOAb		
BDE-28	-0.61 (-1.29, 0.07)	-0.11 (-0.65, 0.43)
BDE-47	-0.49 (-1.09, 0.10)	-0.08 (-0.56, 0.40)
BDE-99	-0.45 (-1.02, 0.11)	-0.24 (-0.71, 0.22)
BDE-100	-0.25 (-0.82, 0.32)	-0.12 (-0.57, 0.33)
BDE-153	-0.0001 (-0.53, 0.53)	-0.23 (-0.67, 0.21)
∑PBDEs	-0.27 (-0.89, 0.34)	-0.15 (-0.65, 0.34)
TgAb		
BDE-28	0.07 (-0.35, 0.50)	-0.02 (-0.45, 0.40)
BDE-47	0.17 (-0.21, 0.54)	-0.01 (-0.38, 0.37)
BDE-99	0.16 (-0.20, 0.51)	0.05 (-0.31, 0.42)
BDE-100	0.08 (-0.27, 0.44)	-0.03 (-0.38, 0.33)
BDE-153	-0.004 (-0.34, 0.33)	-0.001 (-0.35, 0.34)
∑PBDEs	0.12 (-0.26, 0.51)	-0.03 (-0.42, 0.35)

Only includes individuals with complete information for the PBDE exposure and covariates.

[a]Units: PBDEs (ng/g lipid), TPOAb and TgAb (IU/mL). PBDEs were log_{10}-transformed and thyroid antibodies were log-transformed. [b]Adjusted for maternal age, race/ethnicity, education, parity, family income, smoking status, alcohol consumption, gestational age at blood draw, and total serum PCB concentrations. [c]Additionally adjusted for infant gender and mode of delivery.

Table S7. Associations between polybrominated diphenyl ether concentrations and select thyroid hormones in maternal and cord sera by thyroid antibody levels, HOME Study.[a,b]

**Thyroid Hormones by Thyroid Antibody and PBDEs**	**Interaction *p* value**	**PBDEs with TPOAb≤median or TgAb<LOD[c] β (95% CI)**	**PBDEs with TPOAb>median or TgAb≥LOD[d] β (95% CI)**
**Maternal Serum[e]**			
lnTSH			
TPOAb×BDE-28	0.596	-0.15 (-0.57, 0.28)	0.04 (-0.51, 0.59)
TPOAb×BDE-47	0.466	-0.23 (-0.59, 0.14)	-0.01 (-0.47, 0.46)
TPOAb×BDE-99	0.253	-0.25 (-0.59, 0.10)	0.07 (-0.37, 0.52)
TPOAb×BDE-100	0.294	-0.22 (-0.57, 0.14)	0.09 (-0.36, 0.54)
TPOAb×BDE-153	0.227	-0.26 (-0.63, 0.11)	0.07 (-0.30, 0.45)
TPOAb×∑PBDEs	0.165	-0.28 (-0.66, 0.10)	0.16 (-0.33, 0.64)
TgAb×BDE-28	0.895	-0.06 (-0.45, 0.34)	-0.11 (-0.78, 0.56)
TgAb×BDE-47	0.495	-0.18 (-0.51, 0.14)	0.06 (-0.57, 0.68)
TgAb×BDE-99	0.249	-0.19 (-0.49, 0.12)	0.21 (-0.41, 0.84)
TgAb×BDE-100	0.656	-0.13 (-0.45, 0.18)	0.02 (-0.57, 0.60)
TgAb×BDE-153	0.377	-0.03 (-0.34, 0.28)	-0.28 (-0.74, 0.19)
TgAb×∑PBDEs	0.391	-0.18 (-0.51, 0.16)	0.15 (-0.51, 0.80)
TT_4			
TPOAb×BDE-28	0.521	1.09 (0.09, 2.10)	0.56 (-0.75, 1.87)
TPOAb×BDE-47	0.802	0.94 (0.08, 1.81)	0.77 (-0.33, 1.87)
TPOAb×BDE-99	0.501	0.71 (-0.13, 1.54)	0.25 (-0.81, 1.31)
TPOAb×BDE-100	0.616	0.72 (-0.13, 1.56)	0.37 (-0.72, 1.45)
TPOAb×BDE-153	0.258	0.44 (-0.45, 1.33)	-0.30 (-1.20, 0.60)
TPOAb×∑PBDEs	0.461	0.86 (-0.05, 1.78)	0.31 (-0.85, 1.47)
TgAb×BDE-28	0.498	0.71 (-0.22, 1.65)	1.35 (-0.22, 2.93)
TgAb×BDE-47	0.935	0.91 (0.14, 1.68)	0.84 (-0.62, 2.31)
TgAb×BDE-99	0.446	0.69 (-0.05, 1.42)	0.05 (-1.44, 1.55)
TgAb×BDE-100	0.888	0.57 (-0.18, 1.32)	0.46 (-0.95, 1.86)
TgAb×BDE-153	0.998	0.03 (-0.72, 0.77)	0.02 (-1.10, 1.15)
TgAb×∑PBDEs	0.675	0.71 (-0.09, 1.51)	0.34 (-1.23, 1.90)
TT_3			
TPOAb×BDE-28	0.350	12.89 (0.91, 24.87)	3.54 (-12.08, 19.17)
TPOAb×BDE-47	0.281	12.34 (2.01, 22.68)	3.32 (-9.81, 16.45)
TPOAb×BDE-99	0.075*	11.80 (1.94, 21.66)	-2.35 (14.87, 10.17)
TPOAb×BDE-100	0.632	8.57 (-1.51, 18.65)	4.60 (-8.29, 17.49)
TPOAb×BDE-153	0.590	-0.86 (-11.51, 9.80)	3.35 (-7.42, 14.12)
TPOAb×∑PBDEs	0.420	9.40 (-1.56, 20.36)	2.17 (-11.64, 15.98)
TgAb×BDE-28	0.686	8.43 (-2.64, 19.49)	12.93 (-5.72, 31.58)
TgAb×BDE-47	0.676	8.62 (-0.53, 17.77)	12.76 (-4.61, 30.12)
TgAb×BDE-99	0.897	7.42 (-1.23, 16.07)	6.16 (-11.43, 23.75)
TgAb×BDE-100	0.670	5.93 (-2.94, 14.79)	9.96 (-6.54, 26.45)
TgAb×BDE-153	0.865	0.22 (-8.60, 9.03)	1.59 (-11.68, 14.85)
TgAb×∑PBDEs	0.871	6.24 (-3.27, 15.74)	7.95 (-10.51, 26.41)

**Thyroid Hormones by Thyroid Antibody and PBDEs**	**Interaction *p* value**	**PBDEs with TPOAb≤median or TgAb<LOD[c] β (95% CI)**	**PBDEs with TPOAb>median or TgAb≥LOD[d] β (95% CI)**
FT_4			
TPOAb×BDE-28	0.959	0.05 (0.002, 0.10)	0.05 (-0.02, 0.11)
TPOAb×BDE-47	0.786	0.04 (-0.004, 0.08)	0.05 (-0.01, 0.10)
TPOAb×BDE-99	0.831	0.02 (-0.02, 0.06)	0.03 (-0.02, 0.08)
TPOAb×BDE-100	0.931	0.03 (-0.02, 0.07)	0.02 (-0.03, 0.08)
TPOAb×BDE-153	0.492	0.02 (-0.03, 0.06)	-0.01 (-0.05, 0.04)
TPOAb×∑PBDEs	0.814	0.03 (-0.01, 0.08)	0.03 (-0.03, 0.08)
TgAb×BDE-28	0.159	0.03 (-0.02, 0.08)	0.10 (0.02, 0.17)
TgAb×BDE-47	0.048*	0.02 (-0.01, 0.06)	0.11 (0.03, 0.18)
TgAb×BDE-99	0.258	0.01 (-0.02, 0.05)	0.06 (-0.01, 0.14)
TgAb×BDE-100	0.220	0.01 (-0.03, 0.05)	0.06 (-0.01, 0.13)
TgAb×BDE-153	0.855	0.001 (-0.04, 0.04)	0.01 (-0.05, 0.06)
TgAb×∑PBDEs	0.213	0.02 (-0.02, 0.06)	0.07 (-0.004, 0.15)
FT_3			
TPOAb×BDE-28	0.890	0.13 (-0.02, 0.28)	0.15 (-0.05, 0.34)
TPOAb×BDE-47	0.977	0.12 (-0.01, 0.25)	0.12 (-0.05, 0.28)
TPOAb×BDE-99	0.370	0.11 (-0.02, 0.23)	0.02 (-0.14, 0.18)
TPOAb×BDE-100	0.878	0.07 (-0.06, 0.20)	0.08 (-0.08, 0.25)
TPOAb×BDE-153	0.753	-0.01 (-0.15, 0.12)	0.02 (-0.12, 0.15)
TPOAb×∑PBDEs	0.827	0.11 (-0.03, 0.25)	0.08 (-0.09, 0.26)
TgAb×BDE-28	0.307	0.10 (-0.04, 0.24)	0.24 (0.004, 0.48)
TgAb×BDE-47	0.090*	0.08 (-0.04, 0.19)	0.29 (0.07, 0.51)
TgAb×BDE-99	0.190	0.05 (-0.06, 0.16)	0.21 (-0.01, 0.44)
TgAb×BDE-100	0.144	0.03 (-0.08, 0.15)	0.21 (-0.001, 0.42)
TgAb×BDE-153	0.489	-0.02 (-0.14, 0.09)	0.05 (-0.12, 0.22)
TgAb×∑PBDEs	0.134	0.06 (-0.07, 0.18)	0.26 (0.02, 0.49)
**Cord Serum[e,f]**			
lnTSH			
TPOAb×BDE-28	0.218	0.07 (-0.18, 0.33)	-0.18 (-0.48, 0.13)
TPOAb×BDE-47	0.166	0.03 (-0.19, 0.26)	-0.20 (-0.46, 0.06)
TPOAb×BDE-99	0.371	-0.05 (-0.27, 0.17)	-0.20 (-0.44, 0.04)
TPOAb×BDE-100	0.275	0.06 (-0.15, 0.28)	-0.12 (-0.37, 0.13)
TPOAb×BDE-153	0.340	0.14 (-0.08, 0.35)	-0.02 (-0.26, 0.22)
TPOAb×∑PBDEs	0.337	0.03 (-0.21, 0.26)	-0.15 (-0.41, 0.12)
TgAb×BDE-28	0.165	0.07 (-0.18, 0.33)	-0.22 (-0.55, 0.10)
TgAb×BDE-47	0.047*	0.02 (-0.19, 0.24)	-0.34 (-0.63, -0.05)
TgAb×BDE-99	0.074*	-0.06 (-0.26, 0.14)	-0.36 (-0.65, -0.08)
TgAb×BDE-100	0.198	0.04 (-0.17, 0.25)	-0.18 (-0.45, 0.09)
TgAb×BDE-153	0.136	0.17 (-0.05, 0.39)	-0.09 (-0.34, 0.17)
TgAb×∑PBDEs	0.072*	0.04 (-0.18, 0.27)	-0.30 (-0.61, 0.002)
TT_4			
TPOAb×BDE-28	0.743	-0.03 (-0.88, 0.81)	-0.26 (-1.31, 0.79)
TPOAb×BDE-47	0.792	-0.08 (-0.83, 0.67)	-0.24 (-1.12, 0.65)
TPOAb×BDE-99	0.707	0.01 (-0.73, 0.75)	-0.20 (-1.01, 0.62)

**Thyroid Hormones by Thyroid Antibody and PBDEs**	**Interaction *p* value**	**PBDEs with TPOAb≤median or TgAb<LOD[c] β (95% CI)**	**PBDEs with TPOAb>median or TgAb≥LOD[d] β (95% CI)**
TPOAb×BDE-100	0.656	-0.06 (-0.77, 0.66)	-0.30 (-1.14, 0.53)
TPOAb×BDE-153	0.626	0.03 (-0.68, 0.75)	-0.23 (-1.03, 0.56)
TPOAb×∑PBDEs	0.657	-0.04 (-0.82, 0.74)	-0.31 (-1.22, 0.60)
TgAb×BDE-28	0.851	-0.19 (-1.03, 0.66)	-0.06 (-1.13, 1.02)
TgAb×BDE-47	0.695	-0.08 (-0.79, 0.63)	-0.31 (-1.28, 0.65)
TgAb×BDE-99	0.478	0.04 (-0.63, 0.71)	-0.37 (-1.31, 0.58)
TgAb×BDE-100	0.819	-0.22 (-0.91, 0.47)	-0.09 (-0.98, 0.80)
TgAb×BDE-153	0.366	-0.31 (-1.02, 0.41)	0.21 (-0.62, 1.03)
TgAb×∑PBDEs	0.944	-0.15 (-0.89, 0.59)	-0.19 (-1.20, 0.82)
TT_3			
TPOAb×BDE-28	0.998	-4.67 (-12.02, 2.67)	-4.66 (-13.49, 4.17)
TPOAb×BDE-47	0.907	-3.14 (-9.65, 3.37)	-3.72 (-11.26, 3.81)
TPOAb×BDE-99	0.924	-2.28 (-8.70, 4.14)	-2.73 (-9.74, 4.28)
TPOAb×BDE-100	0.620	-2.75 (-8.99, 3.50)	-0.36 (-7.50, 6.78)
TPOAb×BDE-153	0.616	2.96 (-3.25, 9.18)	0.58 (-6.28, 7.44)
TPOAb×∑PBDEs	0.807	-1.43 (-8.20, 5.34)	-2.70 (-10.45, 5.05)
TgAb×BDE-28	0.159	-0.65 (-8.92, 7.62)	-10.34 (-20.89, 0.21)
TgAb×BDE-47	0.371	0.13 (-6.88, 7.13)	-5.16 (-14.68, 4.36)
TgAb×BDE-99	0.564	0.60 (-6.06, 7.26)	-2.64 (-11.88, 6.61)
TgAb×BDE-100	0.744	0.12 (-6.74, 6.97)	-1.74 (-10.57, 7.10)
TgAb×BDE-153	0.700	2.95 (-4.16, 10.06)	0.77 (-7.49, 9.03)
TgAb×∑PBDEs	0.426	1.26 (-6.04, 8.57)	-3.71 (-13.66, 6.24)
FT_4			
TPOAb×BDE-28	0.222	-0.01 (-0.07, 0.06)	-0.07 (-0.15, 0.01)
TPOAb×BDE-47	0.188	0.004 (-0.06, 0.06)	-0.06 (-0.12, 0.01)
TPOAb×BDE-99	0.349	0.02 (-0.04, 0.07)	-0.02 (-0.09, 0.04)
TPOAb×BDE-100	0.161	0.003 (-0.05, 0.06)	-0.06 (-0.12, 0.01)
TPOAb×BDE-153	0.123	0.005 (-0.05, 0.06)	-0.06 (-0.12, 0.0001)
TPOAb×∑PBDEs	0.087*	0.005 (-0.06, 0.07)	-0.08 (-0.14, -0.01)
TgAb×BDE-28	0.658	-0.03 (-0.09, 0.04)	-0.05 (-0.14, 0.03)
TgAb×BDE-47	0.444	-0.02 (-0.07, 0.04)	-0.05 (-0.13, 0.02)
TgAb×BDE-99	0.435	0.002 (-0.05, 0.06)	-0.03 (-0.11, 0.04)
TgAb×BDE-100	0.665	-0.02 (-0.08, 0.03)	-0.04 (-0.11, 0.03)
TgAb×BDE-153	0.893	-0.02 (-0.08, 0.03)	-0.03 (-0.09, 0.04)
TgAb×∑PBDEs	0.269	-0.02 (-0.07, 0.04)	-0.07 (-0.15, 0.01)
FT_3			
TPOAb×BDE-28	0.654	-0.13 (-0.26, -0.001)	-0.08 (-0.24, 0.07)
TPOAb×BDE-47	0.881	-0.08 (-0.20, 0.03)	-0.07 (-0.20, 0.06)
TPOAb×BDE-99	0.961	-0.05 (-0.16, 0.06)	-0.05 (-0.17, 0.08)
TPOAb×BDE-100	0.348	-0.09 (-0.20, 0.02)	-0.01 (-0.13, 0.12)
TPOAb×BDE-153	0.716	-0.02 (-0.13, 0.09)	0.01 (-0.11, 0.13)
TPOAb×∑PBDEs	0.826	-0.08 (-0.20, 0.04)	-0.06 (-0.19, 0.08)
TgAb×BDE-28	0.026*	-0.01 (-0.14, 0.12)	-0.26 (-0.42, -0.09)
TgAb×BDE-47	0.036*	0.01 (-0.10, 0.12)	-0.19 (-0.34, -0.03)
TgAb×BDE-99	0.088*	0.02 (-0.08, 0.13)	-0.13 (-0.28, 0.02)

**Thyroid Hormones by Thyroid Antibody and PBDEs**	**Interaction *p* value**	**PBDEs with TPOAb≤median or TgAb<LOD[c] β (95% CI)**	**PBDEs with TPOAb>median or TgAb≥LOD[d] β (95% CI)**
TgAb×BDE-100	0.199	0.01 (-0.10, 0.12)	-0.11 (-0.25, 0.03)
TgAb×BDE-153	0.828	0.003 (-0.11, 0.12)	-0.02 (-0.15, 0.12)
TgAb×∑PBDEs	0.076*	0.01 (-0.10, 0.13)	-0.17 (-0.32, -0.01)

* p value<0.10

[a]Units: PBDEs (ng/g lipid), TSH (uIU/mL), TT_3 and FT_4 (ng/dL), and FT_3 (pg/mL), TPOAb and TgAb (IU/mL). PBDEs were $\log_{10}$-transformed. [b]Maternal and cord models used antibodies measured in their respective sera. For TgAb: [c] PBDEs with TgAb levels that were not detectable, [d] PBDEs with detectable TgAb levels. [e]Adjusted for maternal age, race/ethnicity, education, parity, family income, smoking status, alcohol consumption, gestational age at blood draw, and total serum PCB concentrations. [f]Additionally adjusted for infant gender and mode of delivery.

Figure S1. β-coefficients and 95% CIs from regression models for associations of BDE-28 and BDE-47 (ng/g lipid) quartiles and thyroid hormones in cord serum. All models adjusted for maternal age, race/ethnicity, education, parity, family income, smoking status, alcohol consumption, infant gender, mode of delivery, gestational age at serum collection, and total serum PCB concentrations. *P* trend was obtained by using the median value in each quartile as a continuous variable in the linear regression models.

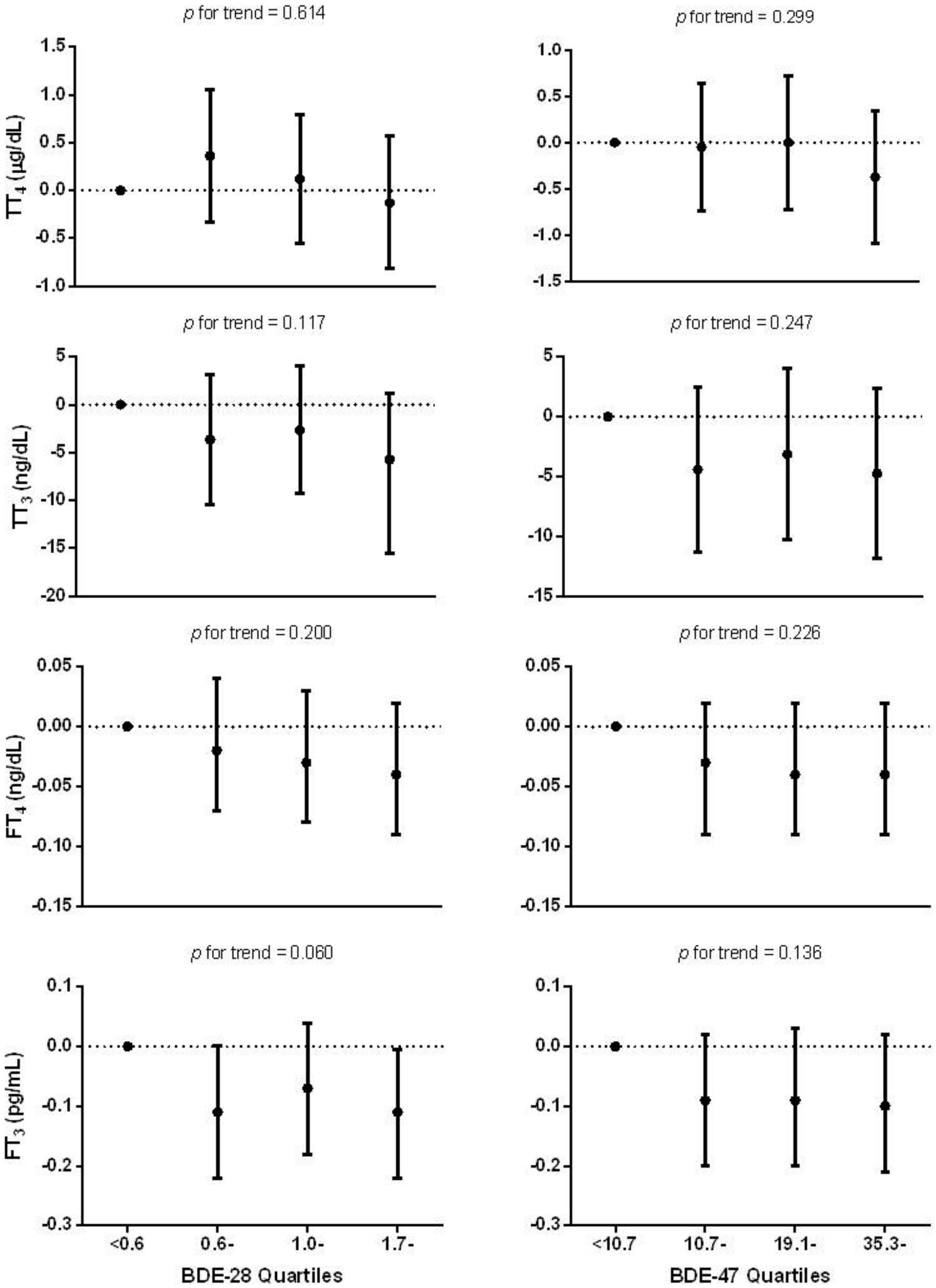

p for trend = 0.614
p for trend = 0.299
p for trend = 0.117
p for trend = 0.247
p for trend = 0.200
p for trend = 0.226
p for trend = 0.060
p for trend = 0.136
TT4 (µg/dL)
TT3 (ng/dL)
FT4 (ng/dL)
FT3 (pg/mL)
<0.6
0.6-
1.0-
1.7-
BDE-28 Quartiles
<10.7
10.7-
19.1-
35.3-
BDE-47 Quartiles

References

Abdelouahab N, Langlois MF, Lavoie L, Corbin F, Pasquier JC, Takser L. 2013. Maternal and cord-blood thyroid hormone levels and exposure to polybrominated diphenyl ethers and polychlorinated biphenyls during early pregnancy. Am J Epidemiol 178:701-713.

Chevrier J, Harley KG, Bradman A, Gharbi M, Sjodin A, Eskenazi B. 2010. Polybrominated diphenyl ether (PBDE) flame retardants and thyroid hormone during pregnancy. Environ Health Perspect 118:1444-1449.

Herbstman JB, Sjodin A, Apelberg BJ, Witter FR, Halden RU, Patterson DG, et al. 2008. Birth delivery mode modifies the associations between prenatal polychlorinated biphenyl (PCB) and polybrominated diphenyl ether (PBDE) and neonatal thyroid hormone levels. Environ Health Perspect 116:1376-1382.

Kim TH, Lee YJ, Lee E, Patra N, Lee J, Kwack SJ, et al. 2009. Exposure assessment of polybrominated diphenyl ethers (PBDE) in umbilical cord blood of Korean infants. J Toxicol Environ Health A 72:1318-1326.

Kim UJ, Lee IS, Kim HS, Oh JE. 2011. Monitoring of pbdes concentration in umbilical cord blood and breast milk from Korean population and estimating the effects of various parameters on accumulation in humans. Chemosphere 85:487-493.

Lin SM, Chen FA, Huang YF, Hsing LL, Chen LL, Wu LS, et al. 2011. Negative associations between PBDE levels and thyroid hormones in cord blood. Int J Hyg Environ Health 214:115-120.

Mazdai A, Dodder NG, Abernathy MP, Hites RA, Bigsby RM. 2003. Polybrominated diphenyl ethers in maternal and fetal blood samples. Environ Health Perspect 111:1249-1252.

Roze E, Meijer L, Bakker A, Van Braeckel KN, Sauer PJ, Bos AF. 2009. Prenatal exposure to organohalogens, including brominated flame retardants, influences motor, cognitive, and behavioral performance at school age. Environ Health Perspect 117:1953-1958.

Stapleton HM, Eagle S, Anthopolos R, Wolkin A, Miranda ML. 2011. Associations between polybrominated diphenyl ether (PBDE) flame retardants, phenolic metabolites, and thyroid hormones during pregnancy. Environ Health Perspect 119:1454-1459.

Zhang J, Jiang Y, Zhou J, Wu B, Liang Y, Peng Z, et al. 2010. Elevated body burdens of PBDEs, dioxins, and PCBs on thyroid hormone homeostasis at an electronic waste recycling site in China. Environ Sci Technol 44:3956-3962.

Zota AR, Park JS, Wang Y, Petreas M, Zoeller RT, Woodruff TJ. 2011. Polybrominated diphenyl ethers, hydroxylated polybrominated diphenyl ethers, and measures of thyroid function in second trimester pregnant women in California. Environ Sci Technol 45:7896-7905.
